# Peptide:glycosaminoglycan hybrid hydrogels as an injectable intervention for spinal disc degeneration†
†The data associated with this paper are openly available from the University of Leeds data repository, http://doi.org/10.5518/47

‡
‡Electronic supplementary information (ESI) available: Full experimental methods, additional self-assembly and gelation data, additional FTIR spectra and TEM images, rheometry frequency sweep plots and peptide purity and content information. See DOI: 10.1039/c6tb00121a



**DOI:** 10.1039/c6tb00121a

**Published:** 2016-05-21

**Authors:** D. E. Miles, E. A. Mitchell, N. Kapur, P. A. Beales, R. K. Wilcox

**Affiliations:** a Institute of Medical and Biological Engineering , University of Leeds , Leeds , LS2 9JT , UK . Email: r.k.wilcox@leeds.ac.uk; b School of Chemistry , University of Leeds , Leeds , LS2 9JT , UK . Email: p.a.beales@leeds.ac.uk; c School of Biomedical Sciences , University of Leeds , Leeds , LS2 9JT , UK; d School of Mechanical Engineering , University of Leeds , Leeds , LS2 9JT , UK; e Astbury Centre for Structural Molecular Biology , University of Leeds , Leeds , LS2 9JT , UK

## Abstract

Degeneration of the spinal discs is a major cause of back pain. During the degeneration process, there is a loss of glycosaminoglycans (GAGs) from the proteoglycan-rich gel in the disc's nucleus, which adversely alters biomechanical performance. Current surgical treatments for back pain are highly invasive and have low success rates; there is an urgent need for minimally-invasive approaches that restore the physiological mechanics of the spine. Here we present an injectable peptide:GAG hydrogel that rapidly self-assembles *in situ* and restores the mechanics of denucleated intervertebral discs. It forms a gel with comparable mechanical properties to the native tissue within seconds to minutes depending on the peptide chosen. Unlike other biomaterials that have been proposed for this purpose, these hybrid hydrogels can be injected through a very narrow 25 G gauge needle, minimising damage to the surrounding soft tissue, and they mimic the ability of the natural tissue to draw in water by incorporating GAGs. Furthermore, the GAGs enhance the gelation kinetics and thermodynamic stability of peptide hydrogels, significantly reducing effusion of injected material from the intervertebral disc (GAG leakage of 8 ± 3% after 24 h when peptide present, compared to 39 ± 3% when no peptide present). In an *ex vivo* model, we demonstrate that the hydrogels can restore the compressive stiffness of denucleated bovine intervertebral discs. Compellingly, this novel biomaterial has the potential to transform the clinical treatment of back pain by resolving current surgical challenges, thus improving patient quality of life.

## Abstract 2

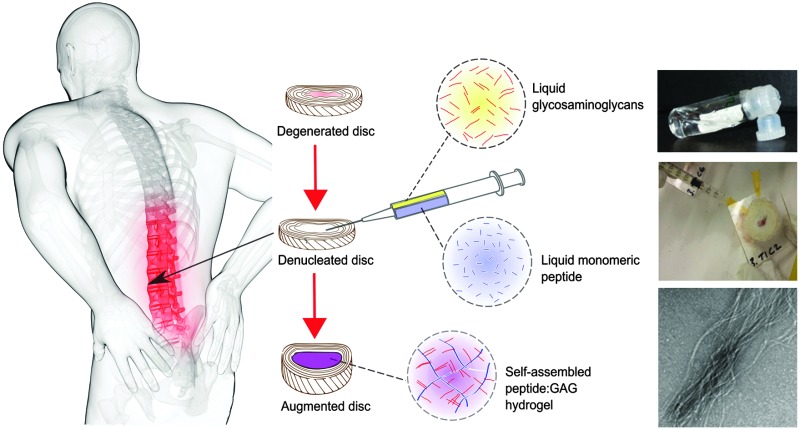
Peptide and glycosaminoglycan hybrid gels undergo self-assembly and result in tuneable mechanical properties with suitability for intradiscal treatments.

## Introduction

Back pain, especially in the lower spine, is strongly associated with degeneration of the intervertebral discs, the soft tissue between the vertebrae that allow their articulation. Current surgical treatments for disc degeneration are highly invasive and have low success rates. With total healthcare and social costs for back pain currently exceeding 1% of GDP in many industrialised nations, there is an urgent social and economic need for more effective therapies that relieve pain and restore the physiological mechanics of the spine.^1–3^

**Fig. 1:**
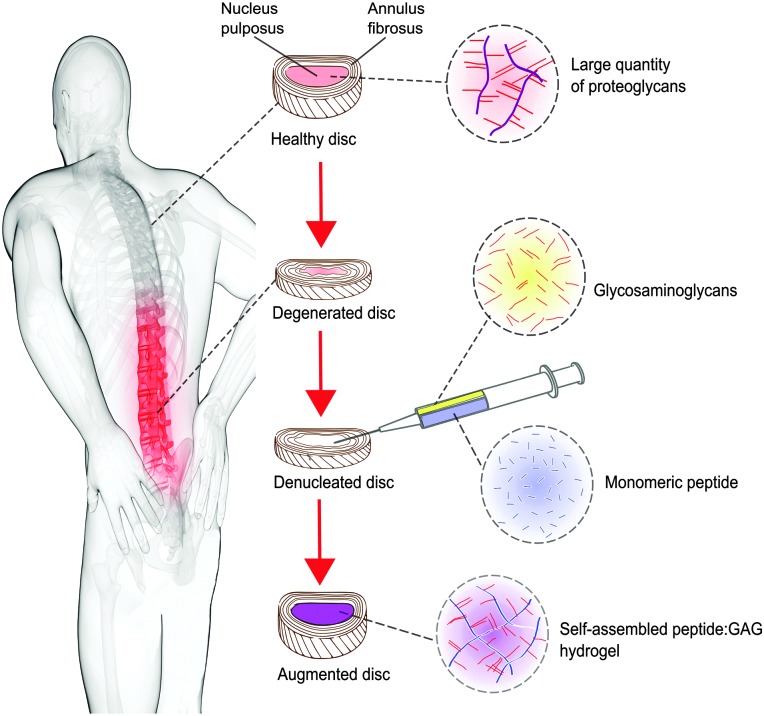
Concept schematic of a peptide:GAG hybrid hydrogel as a minimally invasive nucleus pulposus replacement.

The intervertebral disc is a complex hierarchical structure comprising an outer annulus fibrosus and inner gel-like nucleus pulposus (Fig. 1). The nucleus has a high concentration of proteoglycan macromolecules, which play an important role in the mechanical function of the disc. The glycosaminoglycan (GAG) side chains of the proteoglycans, predominantly chondroitin sulphate, arranged in a comb like structure, provide a fixed negative charge, which causes an influx of small cations into the nucleus. This high salt content results in an osmotic pressure within the disc that provides resistance to compressive loading. One of the main compositional changes during disc degeneration is a decrease in size and quality of the proteoglycan aggregates within the nucleus, which causes the vital glycosaminoglycans (GAGs) to leach out, resulting in a loss of hydration and swelling pressure.^4–6^ It is this reduction in swelling pressure and the consequent changes in the biomechanics of the spine that can cause disc height loss and nerve impingement, ultimately leading to back pain.

One potential therapy to restore biomechanical function to the degenerated disc is to augment or replace the nucleus.^7^ The use of synthetic hydrogels has been explored for this purpose, particularly because their hydrophilic nature can mimic the transport and biomechanical properties of the natural tissue.^8^ However, to date they have had limited clinical success due to their insufficient mechanical properties and extrusion from the treatment site.^7–9^

Self-assembling peptides have many potential biomedical applications.^10–13^ Stimuli-responsive self-assembly and gelation of peptide nanomaterials enables them to be introduced minimally invasively.^14–17^ Furthermore, they can be designed with a wide range of chemical and mechanical properties^18–20^ that could be advantageous for application in nucleus augmentation. Recently, composite hydrogels have been investigated, which aim to supplement or enhance the intrinsic functional properties of these peptide-based materials.^21,22^

The Leeds peptide group have designed a plethora of *de novo* β-sheet tape forming peptides, which self-assemble in one dimension into a hierarchy of well defined structures.^23,24^ They are advantageous because they are based entirely upon natural amino acids and their self-assembly can be triggered by external factors such as pH, ionic strength and temperature.^25–27^

In this work, we investigate whether such a peptide could be optimised for nucleus augmentation applications. Since the function of the intervertebral disc is dependent on the GAG content, we hypothesised that it is possible to mimic the comb-like structures of the natural proteoglycans through non-covalent interactions between chondroitin sulphate chains and the various peptide analogues, therefore optimising the peptides further for this specific application (Fig. 1). To this end, four β-sheet tape forming peptides, systematically varying in charge (net charge of +2*e* or –2*e*) and hydrogen bonding capacity (Glutamine (Q) or Serine (S) based peptides) were selected and combined with GAGs (chondroitin sulphate) (Table 1). The chondroitin sulphate structure has a –2*e* charge and a large number of hydrogen bond donor (4) and acceptor (15) groups per repeat monomer unit that will potentially facilitate interactions between the peptides and these GAGs through a combination of electrostatic and/or hydrogen bonding interactions. Here we demonstrate it is possible to restore the *in vitro* biomechanical performance of the disc by utilising the immense versatility of gel properties that can be achieved. To allow direct comparison of our results with published literature on the self-assembly of P_11_ peptides, the experiments conducted in this report are all undertaken at room temperature; the broad tunability of gel properties that we will demonstrate will allow optimisation of the gel composition for *in vivo* application at 37 °C in future work.

**Table 1:** Peptide and GAG (chondroitin-6-sulphate) structures

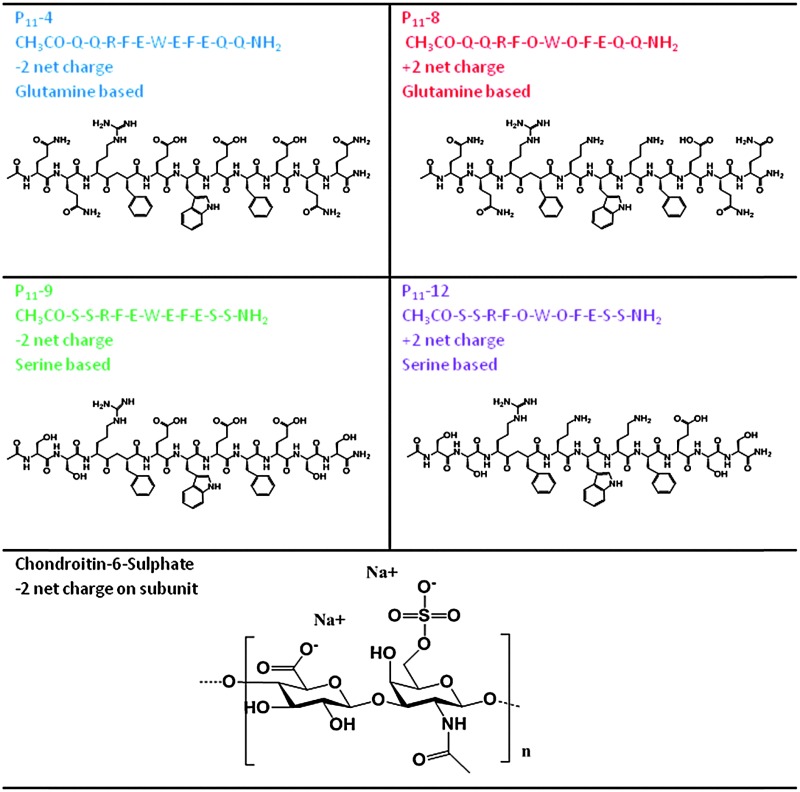

The self-assembly behaviour, mechanical properties and *in vitro* performance of peptide:GAG hybrid hydrogels are reported in this study.

## Results

### The effect of peptide chemistry and GAGs on self-assembly thermodynamics and kinetics

**Fig. 2:**
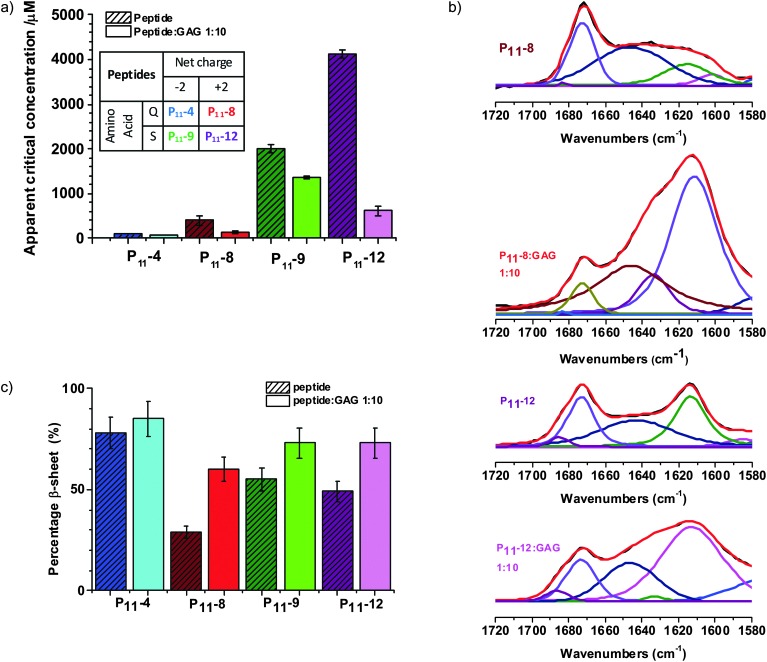
Glycosaminoglycans promote β-sheet peptide fibril formation. (a) Apparent critical concentration for aggregation as determined by ^1^H NMR for peptide only and peptide : GAG 1 : 10 samples, 130 mM NaCl and 1.25 mM TMSP in D_2_O. The table (inset) summarises the net charge and amino acid basis of the peptides. (b) Band fitted FTIR amide I′ region of P_11_-8, P_11_-8 : GAG 1 : 10, P_11_-12 and P_11_-12 : GAG, 1 : 10, red = fitted spectra, black = processed spectra, all other colours = various amide I′ component peaks. (c) Percentage β-sheet as determined by FTIR for peptide only and peptide : GAG 1 : 10 samples in 130 mM NaCl in D_2_O.

We find that in physiological-like conditions the glutamine based P_11_-4 and P_11_-8 have a lower residual monomer concentration than their serine analogues P_11_-9 and P_11_-12 (Fig. 2a). This is in agreement with our previous work that has shown that the thermodynamics and kinetics of peptide self-assembly are strongly dependent upon the peptide's primary structure.^26–28^ Significant to the work reported here, we showed that the glutamine-based peptides had significantly lower critical aggregation concentrations (c*) than their serine-based analogues. We conclude that our selection of β-fibril forming peptide variants will be an important control parameter for the design of the self-assembling hydrogel.

Importantly, we find that the presence of a large amount of GAG does not inhibit P_11_ peptide self-assembly. NMR characterisation of the free peptide monomer concentration that coexists in equilibrium with the peptide fibrils (and therefore the apparent c*) is found to decrease in the presence of GAG (Fig. 2a and Fig. S1a, ESI‡). The cationic peptides exhibit the greater fractional decrease in c*, with the decrease for the serine-based P_11_-12 being most significant.

Significantly for their application as an injectable hydrogel, the GAG also accelerates the self-assembly and gelation kinetics of these peptides. The thermodynamics are affected by the presence of GAG. For example with P_11_-12, the time for the monomer concentration to equilibrate was within two months with the main changes occurring in the first month, whereas with GAG present at a peptide : GAG ratio of 1 : 5, equilibrium was reached in less than a week (Fig. S1c, ESI‡). Visually observed gelation times were also significantly affected. For example for P_11_-8 the time to gel was reduced from days to seconds, even with a low peptide : GAG ratio of 1 : 2, demonstrating the GAG's ability to trigger peptide self-assembly (Fig. S1d, ESI‡). Gelation times were also observed to be significantly reduced from days, hours, or minutes to seconds, with P_11_-12 gelling instantaneously when higher GAG ratios were used (Table S1, ESI‡).

Fourier Transform Infra-Red (FTIR) spectroscopy studies also show that the GAGs do not inhibit the β-sheet peptide secondary structure formation that drives the self-assembly of peptide-only gels (Fig. 2b, c and Fig. S1b, ESI‡). In fact, the GAGs moderately enhance the β-sheet content of the gels, with a larger increase found for the cationic P_11_-8 and P_11_-12 peptides compared to the anionic ones (P_11_-4 and P_11_-9). With GAG present the spectra of all peptides are dominated by a β-sheet band at 1613 cm^–1^ and the weak peak at 1683–1685 cm^–1^ implies anti-parallel arrangement of the β-sheet structure that is consistent with the peptides alone. In all spectra, a broad peak centred around 1645 cm^–1^ coexists with the β-sheet bands suggesting the presence of peptide in non-β-sheet state, which is expected with nucleated self-assembly. A prominent peak centred at 1673 cm^–1^ present in the cationic peptide's spectra is attributed to residual trifluoroacetic acid (TFA) counterions. Therefore the composite hydrogels preserve within them the primary peptide-driven self-assembly interactions that induce the structuring of the pure peptide system. However, while these molecular-scale interactions are conserved, we find significant differences in the hybrid gel structures on microscopic and macroscopic length scales, as we will now show.

### The effect of peptide:GAG interactions on gel microstructure

**Fig. 3:**
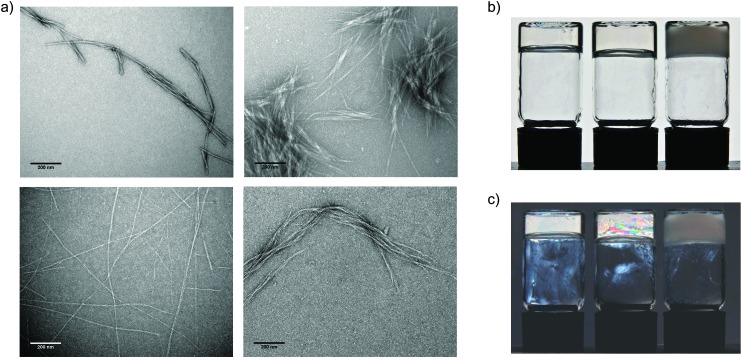
Glycosaminoglycans induce the formation of thicker peptide fibrils. (a) TEM images showing morphology of peptide aggregates, samples with a peptide concentration of 20 mg ml^–1^, 130 mM NaCl in D_2_O, images taken at ×20 000 magnification, scale bars = 200 nm; top left – P_11_-8; top right P_11_-8 : GAG 1 : 10; bottom left P_11_-12 and bottom right P_11_-12 : GAG 1 : 10. (b) Optical micrograph of gel in inverted vial from left to right P_11_-9, P_11_-9 : GAG 1 : 0.5, P_11_-9 : GAG 1 : 10, demonstrating self-supporting nature of gels. (c) Optical micrograph of gel in inverted vial taken through polarised lenses from left to right P_11_-9, P_11_-9 : GAG 1 : 0.5, P_11_-9 : GAG 1 : 10, demonstrating birefringence of gels.

We observe that incorporation of GAGs into peptide hydrogels also modifies their microscopic properties. Transmission electron microscopy images reveal pronounced differences in the fibril morphologies (Fig. 3a and Fig. S2, S3, ESI‡). Broadly speaking, the changes in gel structure between peptide-only and peptide:GAG gels are very similar. In peptide-only gels, the fibril widths are of the order of 10 nm. This compares to peptide:GAG gels where the fibrils align and cluster together with bundle diameters of the order of 100 nm. These results are quantified and presented in Table S2 (ESI‡) for each of the peptides alone and 1 : 10 peptide : GAG mixtures.

Differences in the morphology of GAG-containing gels are also evident on the macroscale. Hydrogels change from transparent GAG-free gels to opaque gels at high GAG : peptide ratios (Fig. 3b), implying the formation of larger aggregate structures within GAG-containing gels that strongly scatter incident light. We also observe the birefringence within these gels at intermediate GAG concentrations, indicating the presence of nematically ordered domains (Fig. 3c). These contrasting characteristics of gel properties in the presence of GAG further confirm the interaction between peptides and GAGs and incorporation of GAGs within these hydrogel structures.

### The effect of peptide:GAG interactions and microstructure on gel mechanics

**Fig. 4:**
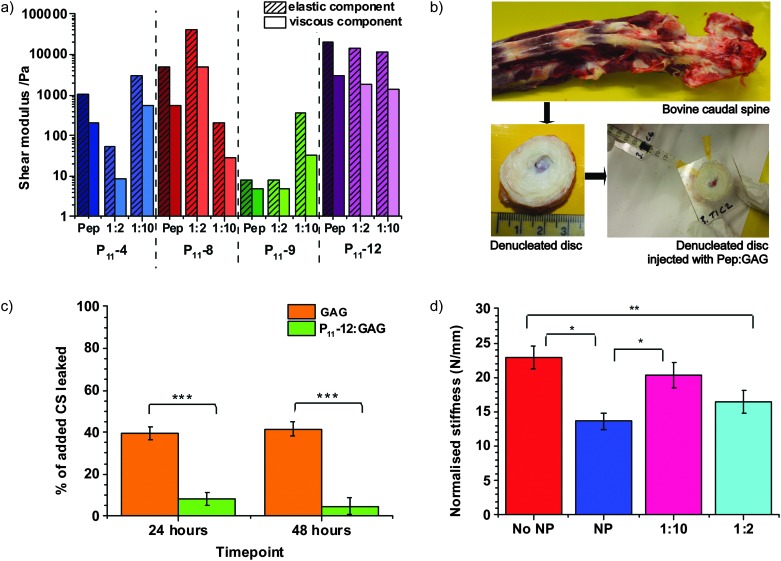
Peptide:GAG hydrogels show potential to restore disc mechanics. (a) Elastic and viscous components of the shear modulus at 2 Hz (walking frequency) for peptide and peptide:GAG samples. (b) Photographs of bovine caudal disc model preparation from the dissection of bovine tail in order to extract discs, removal of the nucleus pulposus and artificial Perspex endplate attachment and final PEP:GAG injection through a 25 G needle with a 25 G needle airhole. (c) Comparison of percentage of added GAG leaked from a disc over a 48 h time period when injected with 1 : 10 GAG only and 1 : 10 P_11_-12 : GAG (error bars = SEM, *n* = 3 one way ANOVA carried out). (d) Plot of normalised stiffness for each sample type as an average of the 6 discs tested No NP = denucleated disc, NP = intact nucleus pulposus, 1 : 10 = denucleated disc augmented with 1 : 10 P_11_-12 : GAG gel and 1 : 2 = denucleated disc augmented with 1 : 2 P_11_-12 : GAG gel. (Error bars = SEM, *n* = 6, one way ANOVA carried out [unless stated difference is non-significant, * ≥95%, ** ≥98%, *** ≥99% confidence that the means are significantly different]).

The specific identity of the peptide and the peptide : GAG ratio are found to be control parameters that tune the gel mechanical properties over an impressive range of four orders of magnitude (Fig. 4a). Rheological measurements of these gels reveal a complex dependence of the elastic and viscous components of the shear moduli on the peptide : GAG ratio. However, for all samples studied, the elastic component is found to be greater than the viscous component, demonstrating the solid-like behaviour of these hydrogels. All peptide:GAG gels show a typical gel profile in the frequency sweep with the phase angle being independent of frequency (Fig. S3, ESI‡).

### The ability of peptide:GAG hydrogels to restore biomechanics

The human gel-like nucleus pulposus has been found to have an elastic component of the shear modulus, *G*′, of approximately 10 kPa and a viscous component of the shear modulus, *G*′′, of approximately 4.5 kPa;^29^ this most closely matches the rheological properties of P_11_-12 1 : 2 and 1 : 10 peptide : GAG ratios. Therefore, these gels were selected for mechanical testing in the more physiological-like conditions of a bovine caudal disc model, although still at room temperature (Fig. 4b). Intervertebral discs are extracted from the bovine tail and their nucleus pulpous is removed. We attach an artificial Perspex endplate to the more cranial disc surface and inject the hydrogel through a narrow 25 G needle, using a second 25 G needle as an airhole to prevent build-up of internal air pressure.

Importantly, we find that we achieve successful *in situ* gelation and the peptide hydrogel significantly reduces leakage of GAGs from the caudal disc model (Fig. 4c). While 39 ± 3% of injected GAG leaches out of the disc after 24 hours when no peptide is present, a 1 : 10 P_11_-12 : GAG hydrogel reduces initial GAG leakage to 8 ± 3%. No further loss of GAG is observed at 48 hours, indicating that most significant GAG outflow occurs in the first day after injection.

As a first step to demonstrating the application of the hydrogel as a minimally invasive therapy for back pain, we compare the normalised stiffness of nucleated discs, denucleated discs and denucleated discs repaired with injected 1 : 10 or 1 : 2 P_11_-12 : GAG hydrogels. We demonstrate that a peptide:GAG hydrogel can restore the biomechanics of a denucleated disc (Fig. 4d). Our data clearly shows that both hydrogels restore the mechanical properties of the disc towards that of a natural intact nucleus (NP). Furthermore the 1 : 2 P_11_-12 : GAG hydrogel restores the biomechanics of a denucleated disc (No NP) to that of an intact nucleus within the error of our measurement.

## Discussion

Self-assembling peptides have potential in the application of nucleus augmentation and to optimise them further they can be mixed with glycosaminoglycans. By mixing them in a ratio where there is an excess negative charge on the GAG chains *e.g.* 1 : 10, 1 peptide with a net overall charge of +2*e* and 10 GAG dimer subunits resulting in an overall net charge of –20*e*, there is a total excess of –18*e* net charge per peptide. This ensures there will be a high concentration of counterions and therefore an osmotic pressure created by the hydrogel.

Our data clearly shows that there is an interaction between the GAG chains and the peptides. This is evidenced by faster gelation kinetics; significantly thicker fibrils observed in the hydrogel and enhanced mechanical properties of these gels.

The presence of GAG in the hydrogels accelerates the self-assembly and gelation kinetics of the P_11_ peptides studied, with the most dramatic effect being on the positively charged peptides where the c* was decreased by over a half. We show that the GAGs enhance the thermodynamic stability of aggregates, increase β-sheet content and can act as a trigger for peptide gelation. We hypothesise, for the positively charged peptides, the anionic GAGs locally concentrate and charge neutralise the peptides in solution, thereby facilitating the assembly of peptide fibrils. However, electrostatic interactions cannot be the full story in explaining favourable peptide–GAG interactions. Other interactions (*e.g.* hydrogen bonding between GAGs and peptides) must play a significant role in order to explain how the GAGs also facilitate the self-assembly of like-charged anionic peptide fibrils (P_11_-4 and P_11_-9).

To confirm that indirect, depletion or excluded volume interactions were not primarily responsible for the observed GAG-induced enhanced gelation, we substituted non-adsorbing polyethylene glycol (PEG) polymers of either 10 kDa or 100 kDa molecular weight at the same mass ratio of peptide : GAG in 1 : 10 gels: we observed no comparable enhancement in gelation kinetics as is found with the GAGs (Fig. S3, ESI‡). Therefore the GAGs must be directly interacting with the peptides *via* physical bonds to enhance their gelation.

We have confirmed that the primary structure of the peptides, which dictates their intrinsic chemistry still plays an important role in the peptide self-assembly with the presence of GAG, in particular the type of polar amino acids. The peptides based on serine side chains exhibit much higher residual monomer concentration and therefore c* values for self-assembly than the peptides based on glutamine side chains. This observation is consistent with the polar zipper effect of glutamines and is unaffected by the presence of GAG.

The presence of GAGs not only affects the peptide interactions but also has an impact on their microscopic structure. The differences in fibril morphologies will be expected to manifest in notable differences in the bulk mechanical properties of these hydrogels. The elastic modulus, or the stiffness of a material, is related to the thickness of the fibrils and/or the number of junction points between interacting fibrils, which in turn is dependent on the density of chains, their width and length and the cross linking affinity at the junction points.^30^ Therefore, if any of these factors are altered, then *G*′ will be altered, so a higher *G*′ suggests more junction points or thicker fibrils and therefore further supports the hypothesis of the peptide and GAG chains interacting.

In order to be a successful therapy for intervertebral disc degeneration, the ideal peptide:GAG hydrogel should have:

• a low c*, resulting in a low level of background monomer present and therefore minimising potential leakage from the treatment site;

• a trigger so that the gelation occurs *in situ* post injection;

• a similar gel stiffness to that of the natural nucleus pulposus;

• a short gelation time to minimise the time of the procedure;

• a high GAG content to mimic the healthy osmotic pressure of the nucleus;

• a good gel stability/lifetime.

The P_11_-12 : GAG 1 : 2 and 1 : 10 gels were chosen as the optimum formulations from these studies because they had good gel stability, a similar elastic modulus to that of the natural tissue and instant *in situ* gelation was possible due to a trigger in the form of the GAG addition. All these attributes are highly desirable for application of these hydrogels in a clinical setting. Although on its own P_11_-12 has a high c* and a long equilibrium time, in the presence of chondroitin sulphate both of these key parameters are drastically reduced.

Following injection into a bovine disc model the presence of P_11_-12 significantly reduced the percentage of GAG leaking out. This confirmed that gelation and self-assembly were successful within the disc and that the P_11_-12 aggregates were interacting with the GAG chains to hold them in place.

The experiment in which the disc models were loaded under static compression was designed in order to gain an initial understanding of how these new peptide:GAG hybrid gels compared to the natural tissue. From the rheology investigation, the P_11_-12 : GAG 1 : 2 gel was expected to restore the most similar biomechanics to the natural tissue as it had the most similar elastic modulus to that of the unconfined nucleus pulposus. Indeed, this is what we observed, with the 1 : 2 gel performing more similarly to the natural nucleus than the 1 : 10 gel. Encouragingly, the specimens augmented with 1 : 2 gel were not significantly different to the intact specimens, and were significantly different from those with the nucleus removed. This restoration of stiffness provides confidence that the peptide:GAG hydrogel has the potential to be a successful intervertebral disc intervention.

For these highly promising injectable hydrogels to progress towards clinical application, several key issues will need to be addressed in future work. Tunability of the gel properties will allow optimisation of the formulation for performance at the physiologically relevant temperature of 37 °C. The long term stability of these hydrogels in relevant physiological environments and hence the practical lifetime of the proposed intervention will also be critical to its success. We will also test its mechanical performance in preclinical simulations that more closely mimic the behavior *in vivo*, for example in a more physiological environment under representative cyclic loading. Such tests should not only measure the compressive stiffness of the disc, but also capture the changes in disc height and annular bulge to gain greater evidence that the gel can provide the necessary biomechanical performance.

## Conclusions

In conclusion, we demonstrate that a peptide:GAG hydrogel can restore the biomechanics of a denucleated intervertebral disc. These hydrogels are injectable through a narrow gauge needle, providing a minimally invasive method of delivering the gel into the nucleus, where the GAG acts as a trigger for self-assembly. We show that the GAG enhances the properties of these hybrid gels by accelerating gelation kinetics and providing thermodynamically more favourable conditions for self-assembly as demonstrated by a significant reduction in c* and, as a result, a significant reduction of free peptide monomer in solution. These novel hydrogels are therefore highly promising biomaterials that could transform surgical practice for the treatment of back pain with substantial benefits for patient prognosis.

## Experimental

Full experimental methods are described in the ESI.‡

### Materials

Peptides were custom synthesised (Polypeptide group, Denmark, NeoMPS, France or CS Bio, USA). Peptide quality control was undertaken by the synthesis company and confirmed in-house (Table 1 and Table S3, ESI‡). The peptide content reflects non-peptide molecules present in the dry peptide mass; these were mainly residual amounts of water, ammonium counterions bound on negatively charged peptide groups and trifluoroacetic acid counterions bound on positively charged groups. All peptides were stored freeze-dried at –20 °C.

Throughout this work the terminology GAG refers to chondroitin sulphate sodium salt from shark cartilage (Sigma Aldrich) and 1 : *n* refers to the molar ratio of one peptide to *n* GAG dimer subunits (*M*_w_ 477).

### Methods

^1^H Nuclear Magnetic Resonance spectra were recorded on a Bruker DPX300 300 MHz spectrometer. Transmission electron microscopy of uranyl acetate stained samples were carried out on a Philips CM10/Jeol JEM1400 electron microscope operating at 80/120 kV. Fourier transform infra-red spectra were acquired with a Thermo Scientific Nicolet 6700 FTIR spectrometer. Rheological measurements used a Malvern Kinexus Pro rheometer with a cone-plate geometry.

Denucleated caudal discs were prepared from the tails of calves less than 30 months old. Perspex endplates were attached with adhesive; samples were injected through a 25 gauge (outer diameter 0.51 mm) needle. Discs were placed in phosphate buffered saline solution after injection and GAG leakage was determined *via* a 1,9 dimethylmethylene blue assay. Static compressive loading experiments were conducted on an Instron 3366 materials testing machine with a 10 kN load cell.
