# Supplementary material for: Peptide:glycosaminoglycan hybrid hydrogels as an injectable intervention for spinal disc degeneration
Source: J Mater Chem B. 2016 May 21;4(19):3225–31. doi: 10.1039/c6tb00121a (PMC4920072; doi:10.1039/c6tb00121a)
Supplement: Supplementary file 1 [file TB-004-C6TB00121A-s001.pdf]

Electronic Supplementary Material (ESI) for Journal of Materials Chemistry B.
This journal is © The Royal Society of Chemistry 2016

Electronic Supporting Information

Peptide:Glycosaminoglycan hybrid hydrogels as an injectable intervention for spinal disc degeneration

Danielle E. Miles, Elizabeth A. Mitchell, Nikil Kapur, Paul A. Beales and Ruth K. Wilcox

Full Experimental Methods

Peptides were custom synthesised (Polypeptide group, Denmark, NeoMPS, France or CS Bio, USA). Peptide quality control was undertaken by the synthesis company and confirmed in-house (**Table 1** and **Table S3**).

The peptide content reflects non-peptide molecules present in the dry peptide mass; these were mainly residual amounts of water, ammonium counterions bound on negatively charged peptide groups and trifluoroacetic acid (TFA) counterions bound on positively charged groups. All peptides were stored freeze-dried at -20°C.

Throughout this work the terminology GAG and CS refers to chondroitin sulphate sodium salt from shark cartilage (Sigma Aldrich) and 1:n refers to the molar ratio of one peptide to n GAG dimer subunits (Mw 477).

All samples stored and experiments carried out at room tempterature.

Proton Nuclear Magnetic Resonance Spectroscopy, ^{1}HNMR

Peptide and peptide:GAG samples were prepared directly in D_2O (Deuterium Oxide, Sigma Aldrich 99.9% D) containing 130 mM NaCl (Sodium Chloride, Fisher Scientific, analytical grade) and 1.25 mM TMSP (deuterated Sodium 3-(trimethylsilyl)propionate-2,2,3,3-d_4, Cambridge Isoptope Labortatories, chemical shift 0 ppm) an internal reference standard. The samples were vortexed for 20 seconds, and then sonicated for 30 minutes. Solution pD was measured and adjusted to 7.4 ± 0.3 using the relation pD = pH + 0.4.[1] Any adjustment of pD, if necessary, was made with µL volumes of 0.1 to 1 mol dm^{-3} DCl (Deuterium Chloride. 35% wt diluted with D_2O, Sigma Aldrich, 99% D) and/or 0.1 to 1 mol dm^{-3} NaOD (Sodium Deuteroxide, 40% wt, diluted with D_2O, Sigma Aldrich, 99% D). Finally, solution vials were closed, sealed with PTFE tape, and then warmed to approximately 80°C to maximise peptide solubility. For the peptide:GAG the chondroitin-6-sulphate solutions were mixed with the peptide solutions while the samples were still warm. The mixture was vortexed again to ensure complete mixing.

NMR data were acquired at room temperature with a Bruker DPX 300 spectrometer, operating at 300 MHz (5 mm probe, spectral width 5995 Hz). A presaturation program was used to minimize the water peak, and 1024 scans were measured per spectrum.

Peptide self-assembly was measured by integrating the splitting pattern of the aromatic region of ^{1}HNMR spectra and normalising with respect to the area of a control peak of TMSP using MestReNova (Mestrelab Research) to process the spectra. In a monomeric state, the coupling interactions between each nucleus is fast and so have well defined line widths, upon, self-assembly the intermolecular freedom decreases and the line widths broaden. Plotting the integral with respect to concentration, the fraction of monomer was determined.

Fourier Transform Infra-Red Spectroscopy, FTIR

Peptide and peptide:GAG samples were prepared as above for ^{1}HNMR but without the addition of TMSP. Samples were placed between CaF_2 crystals and their spectra acquired four days after preparation with a Thermo Scientific Nicolet 6700 FTIR spectrometer. Spectra were averages of 32 scans recorded at room temperature. Blank solvent spectra were subtracted from the sample trace, the baseline corrected and the spectra smoothed. Processed spectra were band fitted in the amide I′ region (1720-1580 cm^{-1}) using the Peak Resolve routine in OMNIC7.3 SP1 (Thermo Electron Corporation), providing information on the number and positions of indivdual component bands. The peak positions corresponding to secondary structures used to determine β-sheet content are presented in the table below:

Amide I' band (cm^{-1})	Secondary struture assignment
1613-1630	β-sheet
1642-1649	Unorderdered
1649-1655	α - helix
1658-1674	Turn
1682-1690	Anti-parallel β-sheet
1694-1697	Turn

In the purification of peptides, trifluoracetioc acid, TFA is used, which leads to it being present in the peptide material as a counter ion bound to the positibvely charged residues. TFA has a FTIR band located at 1673 cm^{-1} and peptides with greater number of arginine and ornithine residues will contain more TFA and therefore a large TFA peak in the FTIR spectra.

Transmission electron microscopy, TEM

P_{11}-9 and P_{11}-9:GAG samples were prepared in DPBS (Dubeccos phosphate buffered saline, Sigma Aldrich), solution containing 0.04% NaN_3, (Sodium azide, Sigma Aldrich, 99%)to prevent bacterial growth. The samples were vortexed for 20 seconds, and then sonicated for 30 minutes. Solution pH was measured and adjusted to 7.4 ± 0.3. Any adjustment of pH, if necessary, was made with µL volumes of 0.1 to 1 mol dm^{-3} HCL (Hydrochloric acid, Fisher Scientific, analytical reagent grade, 37% wt diluted with purified water), and/or 0.1 to 1 mol dm^{-3} NaOH (Sodium hydroxide pellets, Fisher Scientfic, analytical grade, dissolved in and diluted with purified water). Solution vials were closed, sealed with PTFE tape, and then warmed to approximately 80°C to maximise peptide solubility. Following warming GAG powder was added to the peptide solutions and the mixture was vortexed to ensure complete mixing The resulting gels were left to equilibrate for approximately four and a half months.

For P_{11}-4, P_{11}-8 and P_{11}-12, samples with and without GAG prepared for FTIR were used, these were left to equillibriate for 4 days prior to TEM grid preparation.

The gels were then diluted (20 µl of sample in 60 µl D2O) minutes prior to addition to the TEM grids. Glow discharged carbon film coated copper TEM grids (Agar Scientific) were touched onto the peptide and peptide:gag solutions for one minute. The grid was air-dried and then negatively stained with uranyl acetate solution (2% w/v) for 20 seconds and air-dried.

TEM images for P_{11}-4, P_{11}-8 and P_{11}-12, samples with and without GAG were obtained using a Jeol JEM1400 transmission electron microscope operating at 120 kV accererating voltage. TEM images of P_{11}9 with and without GAG were obtained using a Philips CM10 electron microscope operating at 80 kV accelerating voltage.

Rheometry

Peptide and peptide:GAG samples were prepared in DPBS (Dubeccos phosphate buffered saline, Sigma Aldrich), solution containing 0.04% NaN_3 (sodium azide, Sigma Aldrich, 99%). The samples were vortexed for 20 seconds, and then sonicated for 30 minutes. Solution pH was measured and adjusted to 7.4 ± 0.3. Any adjustment of pH, if necessary, was made with µL volumes of 0.1 to 1 mol dm^{-3} HCL (hydrochloric acid, Fisher Scientific, analytical reagent grade, 37% wt diluted with purified water), and/or 0.1 to 1 mol dm^{-3} NaOH (Sodium hydroxide pellets, Fisher Scientfic, analytical grade, dissolved in and diluted with purified water). Solution vials were closed, sealed with PTFE tape, and then warmed to approximately 80°C to maximise peptide solubility. Following warming GAG powder was added to the peptide solutions and the mixture was vortexed to ensure complete mixing The resulting gels were left to equilibrate at room temperature (P_{11}-4 20 days, P_{11}-8 1.5 months, P_{11}-9 one month and P_{11}-12 two months).

Rheometry measurements used a Malvern Kinexus Pro rheometer with a cone-plate geometry (cone angle: 1°, diameter: 50 mm, gap: 0.033 mm). All tests were performed at 25 °C, utilizing a solvent trap and the atmosphere within was kept saturated to minimize evaporation of the peptide samples. The geometery was lowered into position and samples incubated for 15 min.

To ensure measurements were made in the linear viscoselastic regime, amplitude sweeps were performed in a shear strain controlled mode from 0.01-100% at 1 Hz and 20 Hz.

The dynamic moduli of the hydrogels were measured as a frequency function with the sweeps carried out between 1 and 20 Hz.

Bovine disc model preparation

Bovine tails were harvested from calves aged less than 30 months at a local abattoir. To avoid potential damage to the tissue, the discs were stored at 2-8° C prior to experimentation. The tails were cleaned and the spinous processes removed. The discs (C1-C6) were then excised via a transverse cut between the disc and the most cranial cartilage end plate and via a transverse cut through the vertebra, leaving ~10 mm of bone attached to the most caudal side of the disc.

They were placed in monosodium citrate at pH 7.4 for 20 minutes to remove excess blood and through swelling pressures help differentiate the nucleus pulposus from the annulus fibrosus tissue. The discs were then removed from the solution and the nucleus pulposus tissue was excised. The disc and nucleus tissue were weighed prior to and post removal. The discs were then attached to lightly sanded artificial Perspex endplates (2x40x40 mm) using the adhesive Loctite 3090 (Henkel).

For each GAG or Peptide:GAG ratio investigated, discs were chosen from more than one tail over the range of C1 to C6 to try to eliminate variables in disc size, natural GAG levels and tail health. For each group studied, a control of no GAG injection to a denucleated disc was used to determine the average natural GAG leakage over all six tails.

Assessment of GAG leakage through the annulus fibrosus and injection hole

GAG only and peptide solutions were prepared in PBS (phosphate buffered saline solution) and vortexed for 1 min. A total of 250 µl of solution was injected into a disc using a 25 gauge needle (outer diameter 0.51 mm) and syringe with a second 25 G needle as an air hole. For the peptide:GAG augmented discs following an initial injection of 125 µl of the peptide solution, 125 µl of the GAG was injected through the second 25 G needle, retaining the needle used for the peptide injection as a new air hole.

Following injections, the discs were placed in 30 ml of PBS on a orbital shaker for 48 hours. 3 ml aliquots were removed from the surrounding PBS at 24 hours and 48 hours. The 3ml PBS aliquots were analysed via a standard DMMB (dimethylmethylene blue) assay using chondroitin-6-sulphate sodium salt (Sigma Aldrich) to construct the standard curve.

Values are reported as means ± standard error of the mean (SEM), n=3. Results of DMMB assays were analyzed by one-way ANOVA, followed by Tukey's multiple comparison test and were assessed for their statistical significance via paired t-tests (P values < 0.05 were considered as statistically significant).

Static compressive loading

Bovine caudal discs were obtained and prepared as per the above GAG leakage study.

Following preparation, all discs were sealed in individual plastic bags with PBS soaked tissue paper to prevent samples drying and stored at 2-8 ^{o}C overnight. The most caudal vertebra section of each sample was then cast in 70 mm diameter PMMA cement (polymethylmethacrylate, WHW Plastics) to produce a flat surface that was parallel to the Perspex endplate. Once set, the samples were stored in individual sealed plastic bags containing PBS soaked tissue paper at 2-8°C until testing.

All specimens underwent static axial compressive loading from 0 to 9 kN using displacement control at a low load rate of 1 mm/min. The experiments were carried out on an Instron 3366 materials testing machine with a 10 kN load cell (Instron, UK).

It should be noted that at the start of each test, the fixture on the crosshead of the materials testing machine was brought into contact with the Perspex endplate until a load of 0.3 N was recorded. At this point, the displacement and load were zeroed and the test was started.

From the resulting load/displacement plots the normalised stiffness was calculated by multiplying by the specimen height and dividing by its cross-sectional area.

Values are reported as means ± standard error of the mean (SEM), n=6. Results were analyzed by one-way ANOVA, followed by Tukey's multiple comparison test and were assessed for their statistical significance via paired t-tests (P values < 0.05 were considered as statistically significant).

[1] Glasoe, P. K. & Long, F. A. Use of Glass Electrodes to Measure Acidities in Deuterium Oxide. Journal of Physical Chemistry 64, 188-190 (1960).

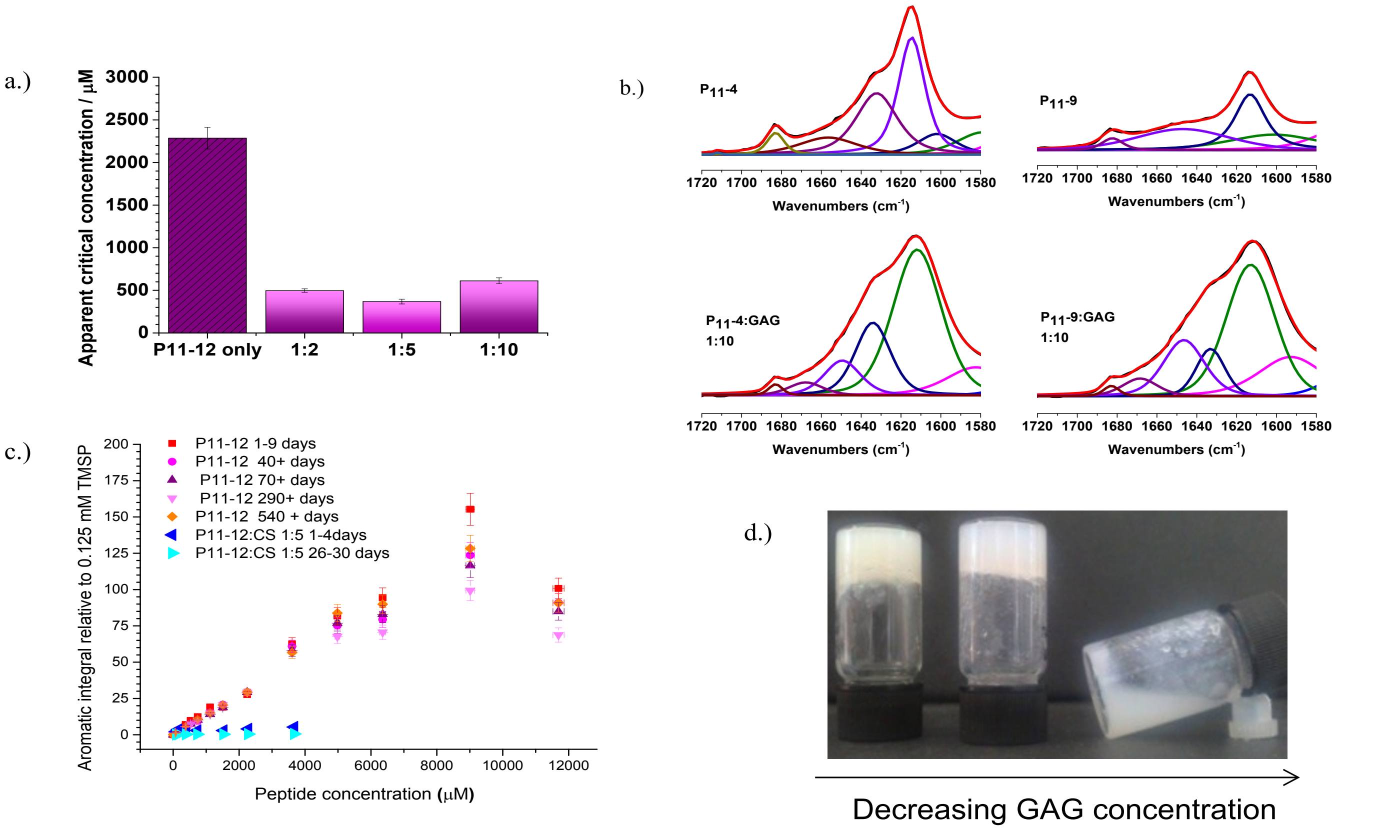

Figure S1. GAG accelerates the self-assembly and gelation kinetics of P_{11} peptides a.) Apparent critical concentration for aggregation as determined by ^{1}HNMR for P_{11}-12 only and P_{11}-12:GAG 1:2, 1:5 and1:10 samples. b.) Band fitted FTIR amide I' bands of P_{11}-4, P_{11}-4:GAG 1:10 , P_{11}-9 and P_{11}-9:GAG, 1:10 c.) ^{1}HNMR aromatic region integral of P_{11}-12 relative to integral of 0.125 mM TMSP reference peak as a function of increasing peptide concentration and time from sample preparation with and without the presence of GAG at a ratio of 1:5. Demonstrating an increase in the kinetics of the system upon the addition of GAG. d.) Image: From left to right P_{11}-8:GAG 1:10, 1:2 and P_{11}-8 only on day of preparation, demonstrating faster gelation kinetics upon addition of GAG.

Peptide	GAG ratio	Time to gel (peptide concentration = 20 mg/ml)
P_{11}-4	Control	Minutes -hours
	1:2	Seconds-minutes
	1:10	Seconds
P_{11}-8	Control	Days
	1:2	Seconds
	1:10	Seconds
P_{11}-9	Control	Minutes
	1:2	Seconds
	1:10	Seconds
P_{11}-12	Control	Minutes
	1:2	Seconds
	1:10	Seconds
	1:50	Spontaneous
	1:100	Spontaneous

Table S1. Typical gelation times in 20mg/ml samples

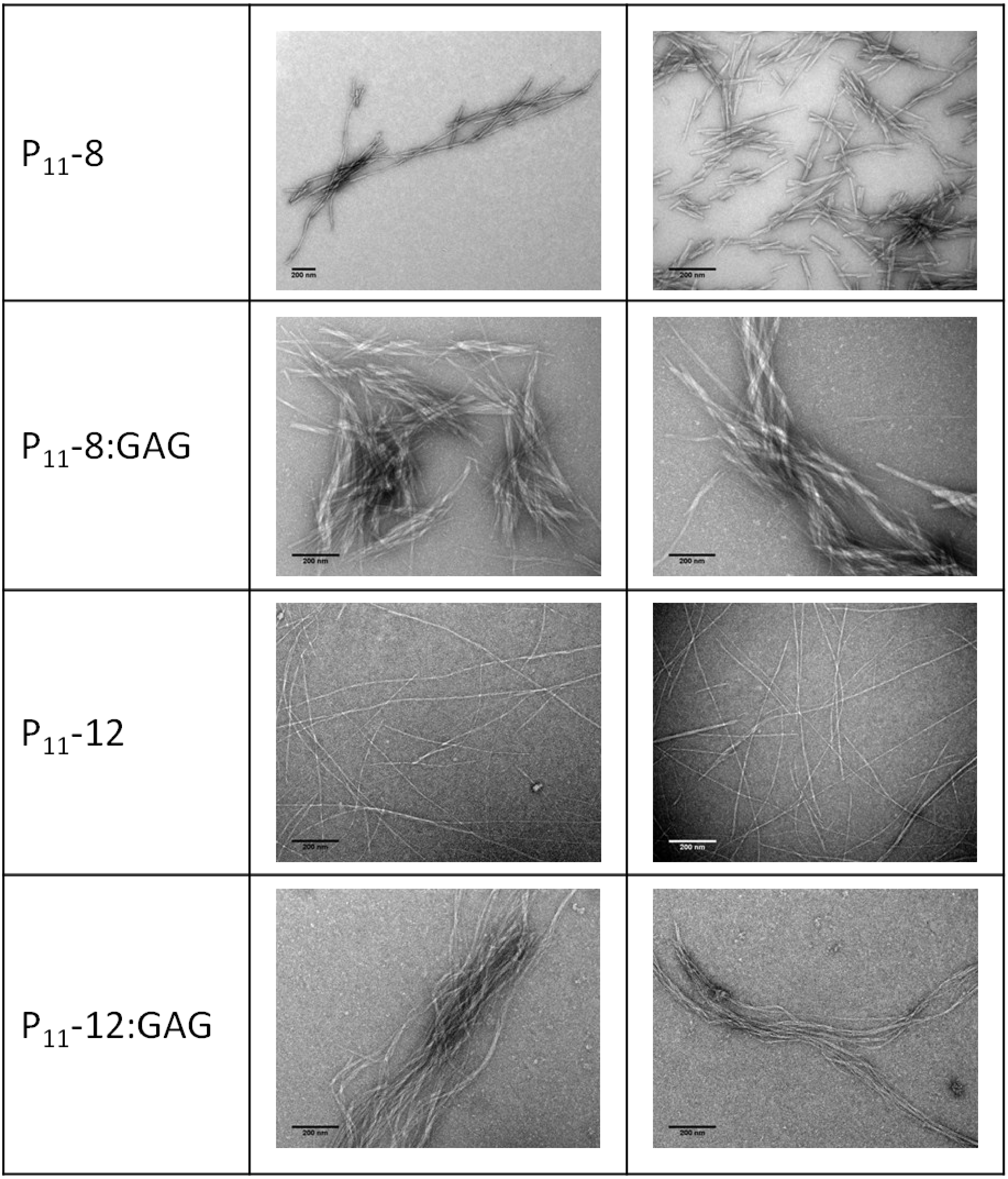

Figure S2. TEM images showing morphology of cationic peptide aggregates, samples with a peptide concentration of 20 mg/ml at 4 days old, images taken at x20,000 magnification, scale bars = 100 nm

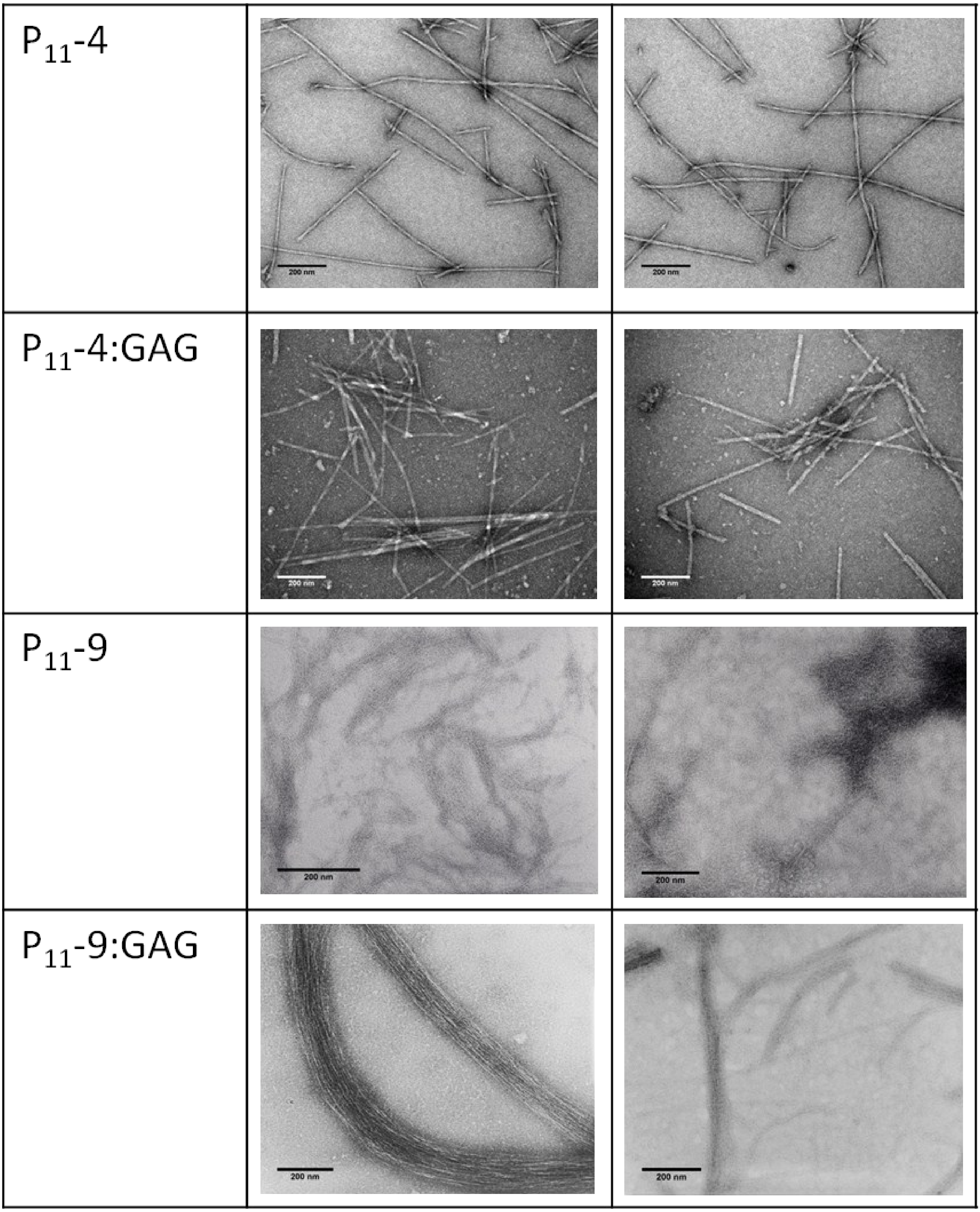

Figure S3. TEM images showing morphology of anionic peptide aggregates, samples with a peptide concentration of 20 mg/ml, images taken at x20,000 (P_{11}-4 and P_{11}-4:GAG), x73,000 (P_{11}-9 right hand side and P_{11}-9:GAG) and 105,000x (P_{11}-9 left hand side) magnification, scale bars = 100 nm

	Fibril width (nm)	Ribbon width (nm)	Twist pitch (nm)	Large loose bundle /fibre width (nm)	Morphology
**P_{11}-4**	14.6 ± 3.7	-	76.6 ± 17.1	-	Twisted fibrils
**P_{11}-4:GAG**	17.5 ± 3.6	-	-	132.4 ± 52	Fibrillar bundles
**P_{11}-8**	13.2 ± 3.3	5.06 ± 1.2	135.2 ± 22.1	-	Twisted fibrils
**P_{11}-8:GAG**	14.5 ± 3.6	-	Fibrils near bundles 1113.2 ± 102.3<br>Fibrils on their own 211.4 ± 18.5	525.7 ± 331.3	Fibrillar bundles
**P_{11}-9**	-	4 ± 1	-	-	Ribbons
**P_{11}-9:GAG**	-	-	-	115 ± 15	Fibrillar bundles
**P_{11}-12**	10.1 ± 2.6	5.0 ± 0.8	114.3 ± 10.3	43.7 ± 11.5	Twisted fibrils and ribbons
**P_{11}-12:GAG**	11.6 ± 2.4	5.2 ± 1.1	-	94.2 ± 26.6	Fibrillar bundles

Table S2. Peptide and Peptide:GAG 1:10 morphology and dimensions of structures as determined by TEM

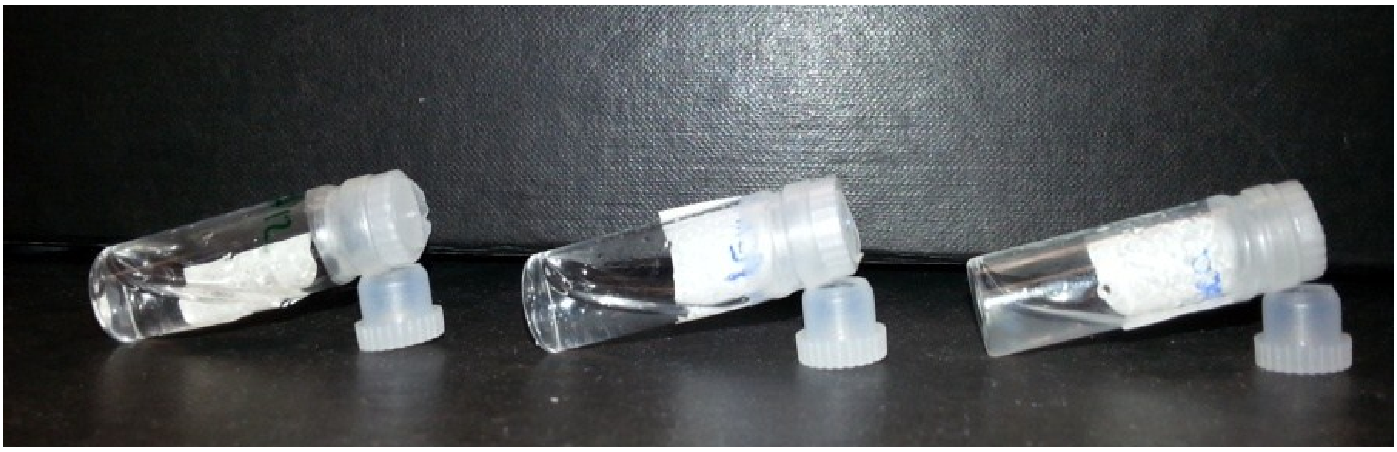

Figure S3. From left to right P_{11}-12, P_{11}-12:PEG (Mw 10k), P_{11}-12:PEG (Mw 100k). 130 mM NaCl in D2O, PEG added at same wt% as 1:10 Pep:GAG ratio. All samples = liquid

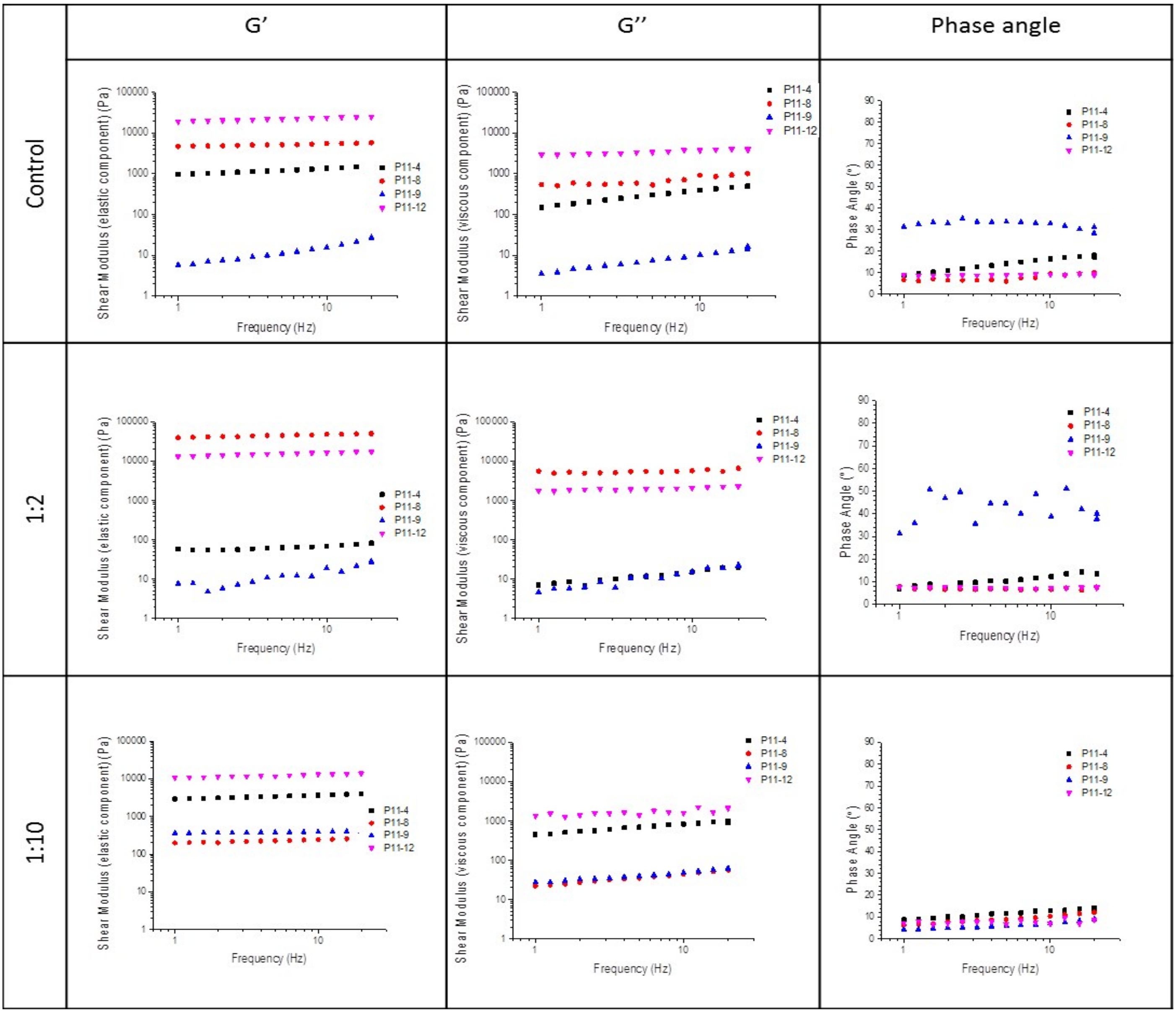

Figure S4. Gel rheological properties of peptide control, peptide:GAG 1:2 and peptide:GAG 1:10, for P_{11}-4, P_{11}-8, P_{11}-9 and P_{11}-12. P_{11}-4 samples 2 months old, P_{11}-8 samples 1 month old, $P1_{1}$-9 samples 2 months old, P_{11}-12 samples 2 months old. As the phase angle is relatively constant for these materials the trend for the viscous and elastic components is similar with an order of magnitude difference.

Peptide	Theoretical mass /Da	HPLC purity /%	Peptide content /%
P_{11}-4	1596	97.3	94
P_{11}-8	1566	96.3	79
P_{11}-9	1432	97 ± 2	92 ± 3
P_{11}-12	1402	97.2 ± 1	74 ± 1

Table S3. Summary of peptide purity and content values across batches for in-house and company supplied data
